# Analysis and Prediction of Urban Surface Transformation Based on Small Baseline Subset Interferometric Synthetic Aperture Radar and Sparrow Search Algorithm–Convolutional Neural Network–Long Short-Term Memory Model

**DOI:** 10.3390/s24082634

**Published:** 2024-04-20

**Authors:** Yuejuan Chen, Siai Du, Pingping Huang, Huifang Ren, Bo Yin, Yaolong Qi, Cong Ding, Wei Xu

**Affiliations:** 1 College of Information Engineering, Inner Mongolia University of Technology, Hohhot 010051, China; chen_yj@imut.edu.cn (Y.C.); 20211800106@imut.edu.cn (S.D.); hwangpp@imut.edu.cn (P.H.); qiyaolong@imut.edu.cn (Y.Q.); 20211100106@imut.edu.cn (C.D.); xuwei1983@imut.edu.cn (W.X.); 2 Inner Mongolia Key Laboratory of Radar Technology and Application, Hohhot 010051, China; 3 College of Resources and Environmental Engineering, Inner Mongolia University of Technology, Hohhot 010051, China; 4 Hohhot Meteorological Bureau, Hohhot 010051, China; 150121a02ih.cdb@sina.cn

**Keywords:** SBAS-InSAR, urban surface deformation, multiple influencing factors, grey correlation analysis, SSA-CNN-LSTM, prediction

## Abstract

With the acceleration of urbanisation, urban areas are subject to the combined effects of the accumulation of various natural factors, such as changes in temperature leading to the thermal expansion or contraction of surface materials (rock, soil, etc.) and changes in precipitation and humidity leading to an increase in the self-weight of soil due to the infiltration of water along the cracks or pores in the ground. Therefore, the subsidence of urban areas has now become a serious geological disaster phenomenon. However, the use of traditional neural network prediction models has limitations when examining the causal relationships between time series surface deformation data and multiple influencing factors and when applying multiple influencing factors for predictive analyses. To this end, Sentinel-1A data from March 2017 to February 2023 were used as the data source in this paper, based on time series deformation data acquired using the small baseline subset interferometric synthetic aperture radar (SBAS-InSAR) technique. A sparrow search algorithm–convolutional neural network–long short-term memory (SSA-CNN-LSTM) neural network prediction model was built. The six factors of temperature, humidity, precipitation, and ground temperature at three different depths below the surface (5 cm, 10 cm, and 15 cm) were taken as the input of the model, and the surface deformation data were taken as the output of the neural network model. The correlation between the spatial and temporal evolution characteristics of the ground subsidence in urban areas and various influencing factors was analysed using grey correlation analysis, which proved that these six factors contribute to some extent to the deformation of the urban surface. The main urban area of Hohhot City, Inner Mongolia Autonomous Region, was used as the study area. In order to verify the efficacy of this neural network prediction model, the prediction effects of the multilayer perceptron (MLP), backpropagation (BP), and SSA-CNN-LSTM models were compared and analysed, with the values of the correlation coefficients of the feature points of A1, B1, and C1 being in the range of 0.92, 0.83, and 0.93, respectively. The results show that compared with the traditional MLP and BP neural network models, the SSA-CNN-LSTM model achieves a higher performance in predicting time series surface deformation data in urban areas, which provides new ideas and methods for this area of research.

## 1. Introduction

In recent years, the process of urbanisation has gradually accelerated, with the year-on-year increase in urban populations leading to the over-exploitation of groundwater, and the increase in the construction of large-scale transport facilities such as subways and viaducts leading to the rapid development of cities, accompanied by the problem of hidden safety hazards. An increasing number of high-risk accidents such as cracks in the urban surface, the collapse of ground buildings, and paralysis of underground systems are occurring, which has seriously affected people’s jobs and daily lives, the safety of life and property, and the environmental protection of cities, and such accidents have become an important influence on the sustainable development of cities [1]. The surface settlement has now become one of the most serious geological issues in Chinese cities, and the accurate prediction of surface deformation in urban areas is of great practical significance for urban planning and disaster warning, management, and control. Therefore, how to accurately monitor, scientifically analyse, and efficiently predict surface deformation in urban areas has become an urgent scientific problem.

Urban ground subsidence is a geological phenomenon that occurs in the process of construction and development; as time passes, human construction activities, the soil structure, and other factors lead to the soil layer not being able to withstand the weight of buildings and their sinking force, which ultimately cause the slow decline of urban surfaces [2]. At present, the methods of urban surface deformation monitoring mainly include level measurements, GPS measurements, total stations, and other equipment and methods, but these have limitations such as high costs, a long observation period, sparse distribution, equipment maintenance, etc., which means that they cannot satisfy the demand for continuous, efficient, and large-area measurements for ground monitoring. With the development of satellite-based radar ground monitoring technology, synthetic aperture radar interferometry (InSAR) has become a constant, all-weather technical means of ground observation. With the rise of time series interferometry techniques, permanent scatterer interferometry (PS-InSAR), which uses highly stable PS point targets to acquire time series deformation information [3], and small baseline subset InSAR (SBAS-InSAR), which uses a small baseline set to acquire time series deformation information [4,5], have been proposed. These methods can overcome the spatial and temporal incoherence and atmospheric delays that are caused by differential interferometric synthetic aperture radar (D-InSAR) techniques to a certain extent, and the measurement accuracy can reach the millimetre level [6], which makes it a promising method for monitoring slow surface deformation over an extended period of time. The development and refinement of the research results of satellite-based monitoring technology will provide a good basis for analysing the spatial and temporal evolution of surface settlement in urban areas. An initial settlement feature extraction and time sequence analysis were achieved using satellite-based monitoring techniques applied to surface settlement in urban areas. Wanwan Zhang et al. [7] used PS-InSAR technology to process two SAR datasets, acquired by the TERRASAR-X and RADARSAT-2 satellites in ascending and descending orbits, respectively, to obtain information on the ground settlement in a typical collapse area in Beijing. Yang Zhang et al. [8] used SBAS-InSAR technology to process RADARSAT-2 satellite data to investigate the relationship between natural and anthropogenic factors and deformation and to obtain information on the settlement of surface deformation in the urban area of Wuhan City, China, from 2015 to 2018. However, in order to accurately understand the characteristics of the spatial and temporal deformation patterns in a study area, it is also necessary to quantitatively analyse the relationship between a variety of variable factors and the evolutionary characteristics of the surface deformation. Surface deformation in urban areas is a cumulative process that is affected by a variety of factors over a long period of time, and there is an intricate causal relationship between human social production and activities, the surface soil layer, and concrete that has been reinforced using temperature, humidity, precipitation, and ground temperature at three different depths below the surface (5 cm, 10 cm, and 15 cm), such as the cumulative effect of the day [9].

The existing methods for urban surface deformation prediction can be classified into three main categories: methods based on physical mechanisms, methods based on mathematical statistics, and methods based on deep learning. The physical mechanism-based approach addresses the physical evolution of subsidence by using modelling and predicting a range of physical parameters that are obtained from fieldwork and measurements. However, the method is subject to tight constraints on the physical parameters and uncertainties such as trends and causes that can lead to a loss of accuracy. The mathematical and statistical methods are based on the analysis of a large number of historical monitoring data, determining intrinsic relationships and trends and through that establishing data models such as regression analysis [10,11], grey prediction [12], and other methods to achieve the simulation of ground subsidence data. However, the method is affected by parametric modelling and -factors, which lead to less accurate prediction results. Deep learning-based approaches take historical monitoring data and their influencing factors and analyse the relationships within the data through neural network prediction models, such as artificial neural networks (ANNs) [13], long short-term memory neural networks (LSTMs) [14], and convolutional neural networks (CNNs) [15]. 

With the rapid development of artificial intelligence and other deep learning technologies, the main research method for predicting surface deformation in urban areas is to improve the accuracy of the prediction through algorithm optimization, combining models, conducting a comprehensive multifactorial influence analysis using algorithms, and variable decomposition and reconstruction of the various prediction methods through analysis of the temporal evolution and the characteristics of surface settlement using a deep learning neural network prediction model to improve the model’s robustness [16]. Ali Radman et al. [17] carried out a surface deformation prediction for Lake Urmia, which is located in the northwest of Iran, with multifactorial inputs using three models: hybrid multilayer perceptron (MLP), CNN, and LSTM. Alternatively, a dynamic model can be built to decompose and reconstruct the dependent variable, and the predictions can be weighted to improve their predictive accuracy. 

Many experts and scholars in the area of multifactorial surface deformation prediction have focused on neural network modelling for the prediction of time series surface deformation data [18]. There are two main categories within this area. Single surface deformation prediction is mainly carried out through the use of various deep learning neural network models to predict the future deformation value in the short term by using the pre-sample data or by using the model’s algorithm to decompose the time series, and then through the neural network. Finally, weighted superposition is carried out to form the final time series deformation value. For example, Cheng Rui et al. [19] set up a VMD-SSA-LSTM model to decompose time series data and then make a comprehensive prediction for the decomposed deformation components and analyse and predict the surface deformation of a mining area. For the prediction of surface deformation in a time series that is affected by a combination of factors, all the influencing factors and deformation data are obtained, and the neural network prediction model is trained by inputting the data from the previous neural network prediction model to obtain the prediction of surface deformation in the short term. For example, yuyi wang et al. [20] used the Erhai Sea region in China as a research object and used a neural network model to predict the surface deformation by combining the four influencing factors in the southern region of the Erhai Sea: built-up areas, water level, cumulative precipitation, and cumulative temperature. 

In this paper, based on the SBAS-InSAR data processing technology, Sentinel-1A satellite images were used to obtain the time series of the surface deformation of Hohhot City. The results show that the deformation trend in Hohhot City is mainly sedimentation in the western and northern regions, while the sedimentation in the eastern region is partially descended. Overall, the regional settlement situation is relatively stable. Then, a grey correlation analysis was used to validate the influence of multiple factors on the surface deformation in urban areas, and a combined prediction model of SSA-CNN-LSTM in urban areas based on the sparrow search algorithm (SSA), a CNN, and an LSTM neural network is presented. The SSA-CNN-LSTM prediction model is proposed to improve the prediction efficiency and accuracy of the neural network model by taking multiple factors as its input variables and searching for the optimal hyperparameters through the SSA algorithm to establish a multifactorial prediction model.

## 2. Data Description

### 2.1. Study Area

**Figure 1:**
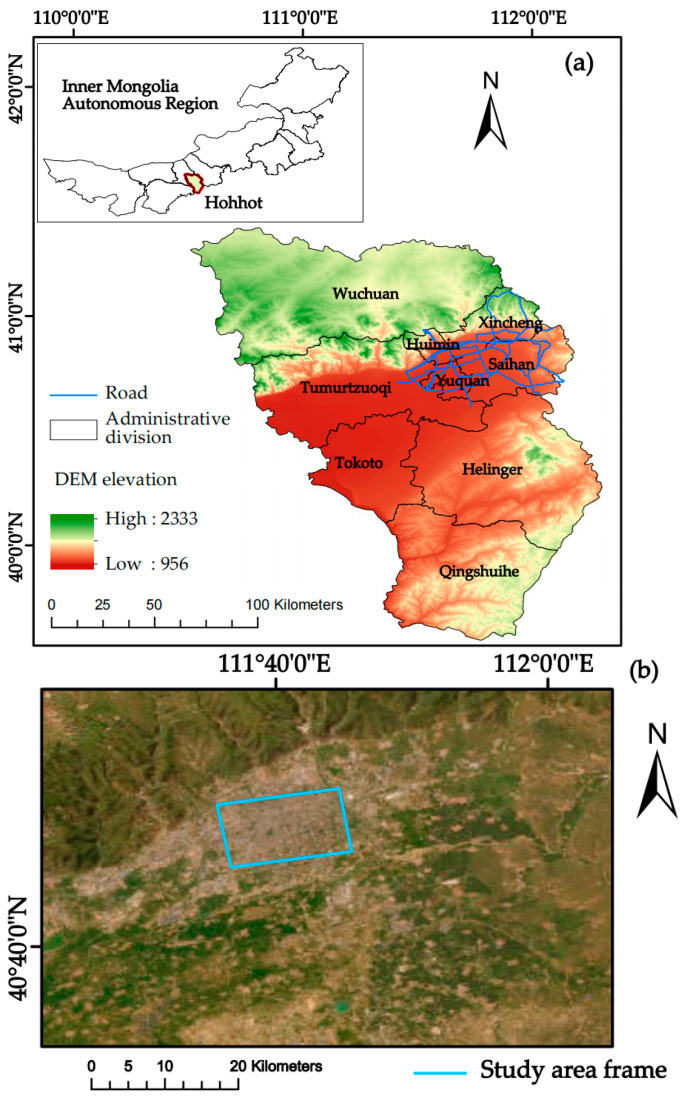
Geographic location and scope of the study area: (**a**) administrative divisions of Inner Mongolia Autonomous Region and Hohhot city; (**b**) surface of research area.

This study was conducted in parts of the urban areas of Hohhot (Saihan, Huimin, Yuquan, and Xincheng districts) in the Inner Mongolia Autonomous Region. Hohhot is the capital city of the Inner Mongolia Autonomous Region, located in the north-western part of China. It has convenient transport links and belongs to the mid-temperate continental monsoon climate, with a large temperature difference and a gradual inclination of the terrain from the north-east to the south-west, with an average annual temperature of 3.5–8 °C, an average annual precipitation of 337–418 mm, and a distinct change of seasons with a pleasant climate. Its geographical location is shown in Figure 1. 

### 2.2. Data

The experimental data involved in this study are as follows: (1) C-band synthetic aperture radar (SAR) images, provided by Sentinel-1A, with a satellite revisit cycle of 12 days/time. The data obtained in this study are the 100-view Hohhot city single-view complex (SLC) image data from Sentinel-1A from 19 March 2017 to 16 February 2023, and the data were obtained from an ASF data search “https://search.asf.alaska.edu/ (accessed on 20 June 2023)”. (2) SRTM DEM, released by NASA, with a ground resolution of 30 m. Precise Orbit Ephemerides (POD) data were obtained from the Copernicus Data Space Ecosystem “https://dataspace.copernicus.eu (accessed on 20 June 2023)”, and the main parameters are shown in Table 1. (3) Meteorological data within the study area of Hohhot City were provided by the Hohhot Meteorological Bureau of the Inner Mongolia Autonomous Region. 

## 3. Methods

### 3.1. Grey Correlation Analysis Based on SBAS-InSAR Deformation Analysis and Using Multiple Impact Factors

In order to prove that the influencing factors will affect the surface deformation of the city to a certain extent, and also indirectly prove that it is feasible to consider multiple factors in a prediction study of surface change, in this paper, we obtained the surface deformation data for the Hohhot urban area by using SBAS-InSAR (ENVI 5.3) technology and used grey correlation to analyse the correlation degree between multiple factors and the surface changes.

**Figure 2:**
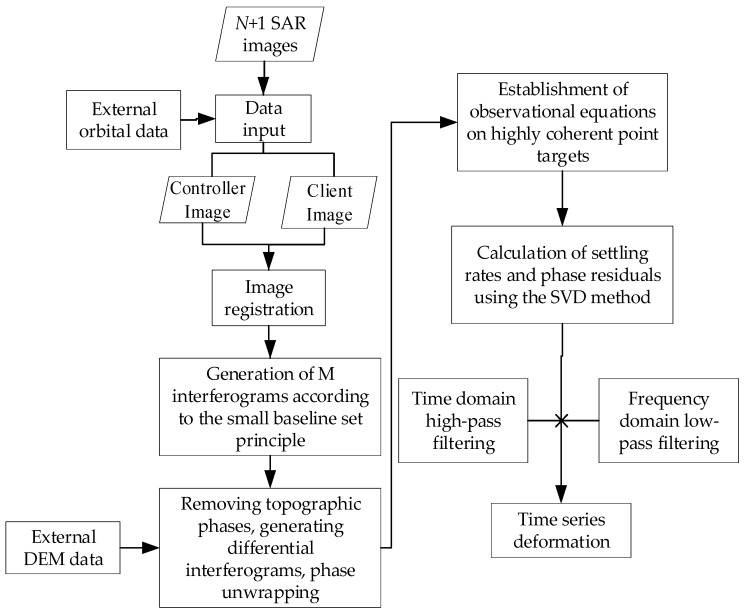
SBAS-InSAR technology processing flow.

The SBAS-InSAR technique [21,22,23,24] performs deformation measurements by means of a small baseline differential interferometric atlas, which can reduce the influence of spatial de-correlation and terrain errors, and then applies the singular value decomposition (SVD) method based on the least-paradigm criterion of the deformation rate to obtain the deformation rate of coherent targets and their time series [25]. Its technological flow is shown in Figure 2.

**Figure 3:**
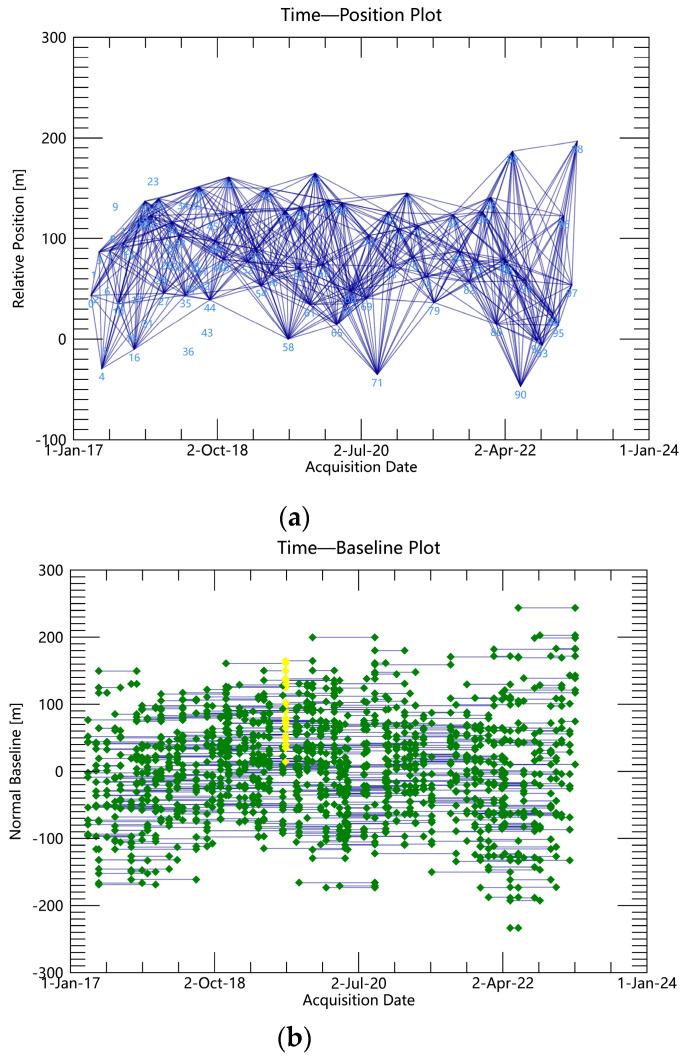
Generation of small baseline sets: (**a**) temporal baseline; (**b**) spatial baseline.

**Figure 4:**
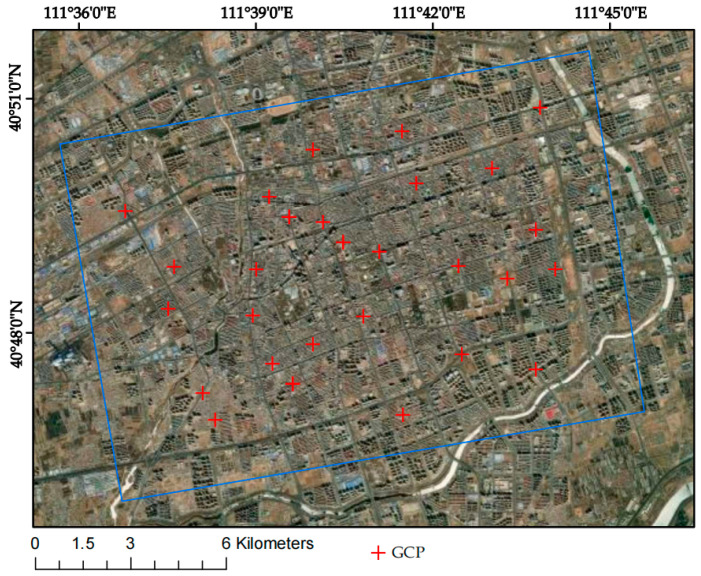
GCP reference point.

**Figure 5:**
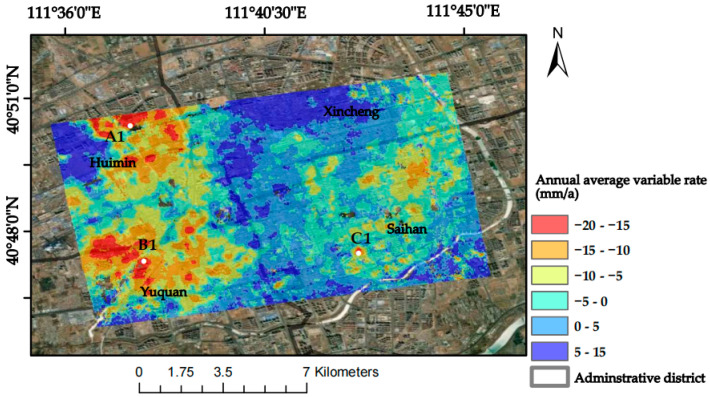
SBAS-InSAR annual mean deformation rate and location of subsidence centres.

In this paper, SBAS-InSAR processing is performed on 100-view Sentinel-1A radar image data [26,27]. The specific data processing steps are as follows:

(1) Interferometric connectivity map generation. By means of interferometric image pair pairing of 100-view image data, the 15 January 2020 image is selected as the super-controller image, the SAR image is optimally combined to form a short baseline set, and the generated spatio-temporal baseline condition is shown in Figure 3a,b. The interferogram controller sub-image is sorted in chronological order to obtain the controller–client image sequence, $IE=[IE_{1},\cdots ,IE_{M}]$, $IS=[IS_{1},\cdots ,IS_{M}]$, and $IE_{k}>IS_{k},\forall k=1,\cdots ,M$; all the differential interferograms can be combined into a system of observation equations:
  (1) $$\delta \phi_{k}=\phi (t_{IE_{k}})-\phi (t_{IS_{k}}),\forall k=1,\cdots ,M$$
(2) Differential interference workflow. After the aligning, deplaning, and topographic phase processing of interfering pairs, adaptive filtering is used to consistently phase out noise and obtain smooth differential interferograms. Then, phase de-entanglement is carried out using the least-cost flow method, and interfering pairs with poor coherence or de-entangled phase transitions are rejected and re-decomposed.
(3) Orbital refinement and re-delevelling. A reference point (the GCP point) is selected as the orbit refinement control point, and the GCP point is used to estimate and remove the residual constant phase, as well as the phase ramps that remain after unwrapping. The GCP reference point is shown in Figure 4.
(4) Deformation rate and DEM coefficient estimation. The differential phase of any image element (x, y) on the kth interferogram can be simplified by using a polynomial model to assist the external DEM for orbit refinement and re-deplaning, removing the terrain phase and ignoring the noise and other phases (see Equation (2)). The deformation and elevation information of all image pairs are inverted based on the linear model to estimate the deformation rate and residual terrain phase. This is shown in Equation (3).
  (2) $$\begin{array}{ll} \delta \phi_{k}(x,y) & =\phi (t_{B},x,y)-\phi (t_{A},x,y)\\ & \approx \frac{4\pi }{\lambda }[d(t_{B},x,y)-d(t_{A},x,y)] \end{array}$$
  (3) $$v^{T}=[v_{1}=\frac{\phi_{1}}{t_{1}-t_{0}},\cdots \cdots ,v_{N}=\frac{\phi_{N}-\phi_{N-1}}{t_{N}-t_{N-1}}]$$
(5) Atmospheric phase and terrain residual phase removal. Atmospheric delayed phases are estimated and removed using spatio-temporal filtering, and orbital error residual phases are updated to separate line-of-sight deformation information, as shown in Equation (4).
  (4) δφ=Bv
(6) Geocoding. Geocoding the SBAS results after atmospheric correction, the final deformation rate information is obtained for 539,184 points, and the obtained surface deformation results are shown in Figure 5.

PS-InSAR is a surface deformation monitoring technique that uses multi-scene SAR imagery of the same area to analyse scattering points that are coherent and stable over time, in terms of time series amplitude and phase, and are not easily affected by spatial and temporal incoherence factors [28]. Through the main and auxiliary imaging standards, differential interference SAR image processing uses the primary digital elevation model (DEM) to remove the terrain phase, generate the interference atlas, use the amplitude separation index to select the candidate point and use the three-dimensional diluted separation network to solve the phase. The nonlinear deformation and the atmosphere are separated through time- and space-domain filtering, and finally, the millimetre-level surface transformation information is obtained through geographical coding.

**Figure 6:**
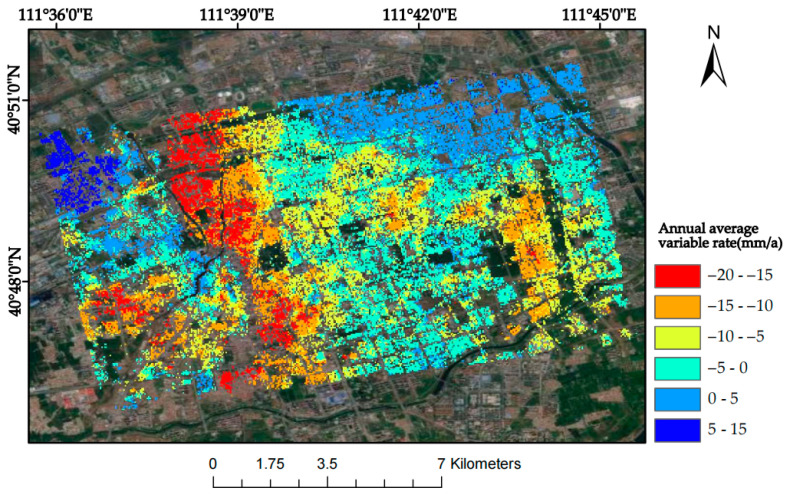
PS-InSAR annual mean deformation rate and location of subsidence centres.

**Figure 7:**
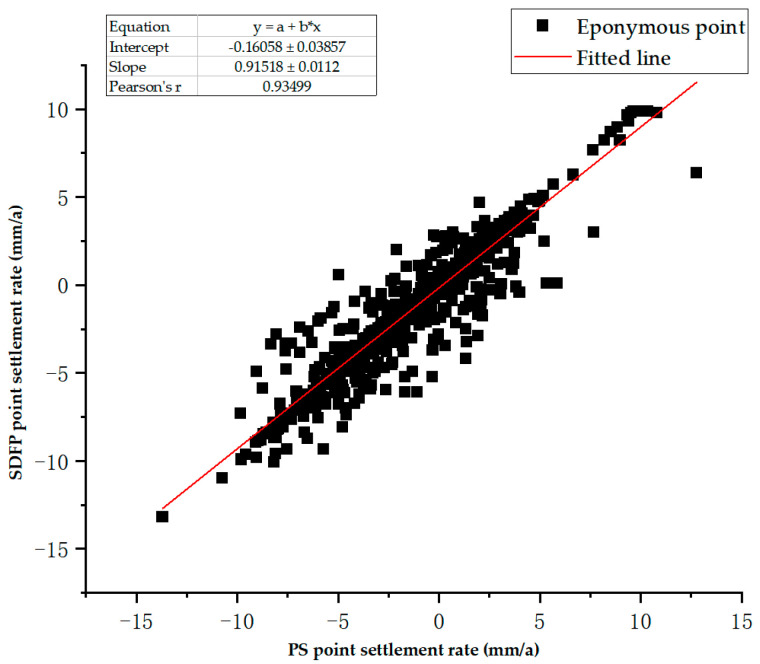
Correlation analysis plot between PS-InSAR and SBAS-InSAR.

In order to verify the correctness of the InSAR results, the PS-InSAR technology obtains the surface transformation data of the research area, and the 963 settings of the same name are selected to compare the data with SBAS-InSAR for comparative verification. The surface transformation of the PS-InSAR technology is shown in Figure 6, and the correlation analysis plot between PS-InSAR and SBAS-InSAR is shown in Figure 7.

The results from the above comparison analysis prove that the surface transformation data that were obtained using SBAS-InSAR technology in the research area are reliable.

Grey correlation analysis is based on grey correlation by comparing the degree of similarity between the geometric relationship of the data series and the geometric shape of the curve. It is an analytical method that can be used to analyse the degree of correlation between factors of a system by assessing the relative strength of the influence of a certain item compared with the other factors in the grey system [29]. The key steps are as follows:

Determine the multi-column matrix data that affect the behaviour of the system, as shown in Equation (5).

(5) $$X_{2}=\left[\begin{array}{c} X_{0}\;\;\;\;X_{1}\;\;\;\;\cdots \;\;\;\;X_{n} \end{array}\right]=\left[\begin{array}{cccc} x_{0}(1) & x_{1}(1) & \cdots & x_{n}(1)\\ x_{0}(2) & x_{1}(2) & \cdots & x_{n}(2)\\ \vdots & \vdots & & \vdots \\ x_{0}(m) & x_{1}(m) & \cdots & x_{n}(m) \end{array}\right]$$

To exclude the impact of the difference between the units of each indicator and their value in the order of magnitude of the disparity that is brought about by the phenomenon of irrationality, reduce the absolute value of the data differences. A dimensionless matrix holds the initial value in this method, as well as the average value, etc., and in this paper, the initial value of the dimensionless matrix is calculated as shown in Equation (6).

(6) $$X_{i}(k)=\frac{X_{i}^{\prime }(k)}{X_{i}^{\prime }(1)}$$

Calculate the difference sequence, the absolute difference between the corresponding elements of each evaluated object’s indicator sequence (comparison sequence), and the pre-reference sequence, as shown in Equation (7).

(7) $$\begin{array}{l} \Delta_{i}(k)=\left|X_{0}^{\prime }(k)-X_{i}^{\prime }(k)\right|\\ \Delta_{i}=(\Delta_{i}(1),\Delta_{i}(2),\cdots ,\Delta_{i}(n)),i=1,2,\cdots ,n \end{array}$$

Calculate the maximum and minimum difference between the two levels, as shown in Equation (8).

(8) $$\begin{array}{l} m=\min_{i=1}^{n}\min_{k=1}^{m}\left|X_{0}(k)-X_{i}(k)\right|\\ M=\max_{i=1}^{n}\max_{k=1}^{m}\left|X_{0}(k)-X_{i}(k)\right| \end{array}$$

Calculate the grey correlation coefficient, as shown in Equation (9).

(9) $$\zeta_{i}(k)=\frac{\min_{i=1}^{n}\min_{k=1}^{m}\left|X_{0}(k)-X_{i}(k)\right|+\rho \max_{k=1}^{m}\left|X_{0}(k)-X_{i}(k)\right|}{\left|X_{0}(k)-X_{i}(k)\right|+\rho \max_{i=1}^{n}\max_{k=1}^{m}\left|X_{0}(k)-X_{i}(k)\right|}$$

where k=1,2,⋯m, i=1,2,⋯,n, ρ is the discrimination coefficient of the degree of association between the balanced indicators of the model, using values within (0, 1). The larger the value is, the smaller the difference between the coefficients of association is, and the weaker the ability to distinguish, generally 0.5, will be.

To calculate the grey correlation degree, since the grey correlation coefficient ($r_{i}$) is used to evaluate the degree of relationship between different levels of each factor and the standard indicators, the difference in the grey correlation coefficients of different levels of the same factor results in a more dispersed evaluation of the factor’s degree of influence on the system as a whole [30]. Therefore, the average value of the grey correlation coefficients at different levels of the factors is usually taken to be the quantitative value for evaluating the degree of association between the original factor columns and the standard columns, as shown in Equation (10).

(10) $$r_{i}=\frac{1}{m}\sum_{k=1}^{m}W_{k}\zeta_{i}(k),k=1,2,\cdots ,m$$

### 3.2. Surface Deformation Prediction Model Based on SSA-CNN-LSTM Neural Network

**Figure 8:**
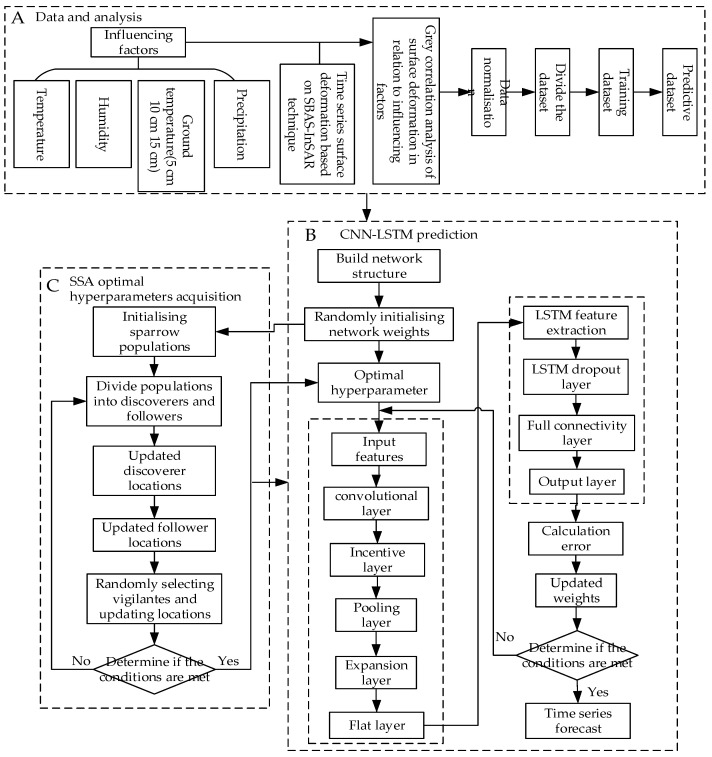
SSA-CNN-LSTM implementation process: (**A**) stands for the data preparation stage, (**B**) stands for the CNN-LSTM prediction stage, and (**C**) stands for the SSA hyperparameter acquisition stage.

The SSA-CNN-LSTM model is a multi-influence surface deformation prediction model constructed by using the SSA, CNN, and LSTM neural network [31,32]. Combining the fast optimisation parameter search of the SSA, the strong feature information extraction function of the CNN, and the short-term fine prediction capability of the LSTM model, the optimal parameter values are searched for by the SSA. Ultimately, the purpose of minimising the prediction value error of the CNN-LSTM model is achieved, and the prediction accuracy is improved. As shown in Figure 8, the specific steps of the SSA-CNN-LSTM neural network prediction model are as follows:

(1) Data preparation and analysis stage: The SBAS-InSAR technique was used to acquire time series surface deformation data in the study area. The air temperature, humidity, precipitation, and ground temperature at 5 cm, 10 cm, and 15 cm below the surface in the study area were used as input data, and the time series cumulative surface deformation data at the feature points were used as the output. The acquired data were normalized to remove the effect of magnitude, and the data were chemically divided, including 70 groups for the training set, 21 groups for the test set, and 9 groups for the validation set.
(2) SSA parameter optimisation phase: Initialise the number of sparrow populations, update the sparrow population position, determine the quantitative evaluation method of adaptation, and calculate the adaptation value and the adaptation function. Input the data into the CNN-LSTM network, construct the network structure, randomly initialise the network weights as inputs for the SSA algorithm, initialise the sparrow population, divide the population into discoverers and followers, and then under the influence of the optimal search of the network hyperparameters, continuously update the network by updating the discoverer position and follower position. Update the randomly selected vigilantes and update the position to determine whether the optimal hyperparameter iteration stopping condition is satisfied. If so, obtain the optimal hyperparameters of the CNN-LSTM network. Otherwise, go back to the division of the population again. The SSA is used to obtain the optimal learning rate, number of hidden nodes, and regularisation coefficients and constraints in the CNN-LSTM model. Among them, the parameters of the sparrow search algorithm are shown in Table 2.
(3) Predictive modelling stage: Randomly initialise the network weights and transfer the optimal network hyperparameters that were obtained by means of the SSA to the CNN-LSTM model, which decodes these to obtain the optimal values of the learning rate, hidden nodes, and regularisation coefficients. The optimised convolutional layer performs feature extraction on the input data, inputs them into the LSTM layer for feature extraction, updates the weights of the entire neural network model, determines whether it meets the accuracy requirements or reaches the maximum number of iterations, and if it does not meet the iterative stopping conditions, then the convolutional layer returns to the parameter optimisation step and re-processes to ensure that it meets the conditions and ultimately obtains the timing prediction results. The main SSA-CNN-LSTM parameter settings are shown in Table 3.

The SSA algorithm can be used to control the movement position between the sparrow and the food according to the fitness value, which increases with the number of iterations, thus achieving an iterative optimisation of the food structure and the optimal solution of the global problem. Using this approach, the final optimised network model and parameters can be obtained by passing different input data through the convolutional and LSTM layers, respectively. The CNN extracts the features from different input data, while the LSTM layer learns the features that have been extracted by the CNN and then inputs the output features into the fully connected layer for prediction. The training set that is obtained from data processing is used for network simulation training, and finally, the test set data are predicted to derive the error between the predicted output value and the actual value. In this process, the value of the SSA fitness function is continuously reduced to minimise the mean square error with the increase in the number of iterations, and the resulting network model is optimal, which in turn minimises the error between the predicted value and the true value [33,34].

The SSA is a global optimisation algorithm based on the foraging and anti-predation activities of sparrow populations [19]. It has significant advantages in terms of convergence speed and convergence accuracy. The basic idea of the sparrow search algorithm is to optimise the parameters by dividing the population, updating the guidance mechanism for the population’s location, determining the optimal food source according to the fitness function, and finally, determining the global optimal parameters.

Discoverers provide a foraging direction for the population and guidance for followers, and the location updates are shown in Equation (11).

(11) $$u_{i,j}^{t+1}=\left\{ \begin{array}{ll} u_{i,j}^{t}\cdot \exp(-\frac{i}{\alpha \cdot t_{\max}}) & Q<R\\ u_{i,j}^{t}+A\cdot S & Q\geq R \end{array}\right.$$

where t is the number of iterations, $t_{\max}$ is the preset maximum number of iterations, $u_{i}^{t}$ and $u_{i}^{t+1}$ denote the position of the sparrow at time t and at time t + 1, respectively, after the update, α is a random number in [0, 1], and R∈[0.5,1] is the alarm threshold. When Q<R, it indicates that the sparrow is discovering, entering search mode, and updating its location. When Q>R, it indicates that the vigilant in the population has detected a danger and the discoverer stops foraging at this time and flies to a safe location.

The identities of discoverers and followers will shift, but the proportion of both in the population is fixed, there are no predators in the foraging environment, and the producers can perform the optimal search and provide optimal guidance to the joiners. The first m sparrows with high adaptation in each generation are discoverers, *n*-m sparrows are followers, and the follower’s position is updated as shown in Equation (12).

(12) $$v_{i,j}^{t+1}=\left\{ \begin{array}{ll} A\cdot \exp(\frac{v_{worst}^{t}-v_{i,j}^{t}}{i^{2}}) & i>\frac{n}{2}\\ v_{i,op}^{t+1}+\left|v_{i,j}^{t}-v_{i,op}^{t+1}\right|\cdot \left[B^{T}(BB^{T})\right]S & others \end{array}\right.$$

where $v_{worst}$ is the worst position in the global search of the population, $v_{i,op}$ is the optimal position in the population at the current moment, and B is a dimensional matrix with elements with random values of −1 or 1.

In the sparrow population, the initial positions of all sparrows are randomly generated. Assuming that a small percentage of sparrows will be aware of the danger and will quickly move closer to their surrounding companions to reduce the risk of their own predation, the position of this category of sparrows is updated as shown in Equation (13).

(13) $$w_{i,j}^{t+1}=\left\{ \begin{array}{ll} w_{best}^{t}+\beta \cdot \left|u_{i,j}^{t}-u_{best}^{t}\right| & f_{i}>f_{b}\\ u_{i,j}^{t}+C\cdot \left(\frac{w_{i,j}^{t}-u_{worst}^{t}}{(f_{i}-f_{w})+D}\right) & f_{i}<f_{b} \end{array}\right.$$

where $w_{best}^{t}$ is the global optimal position at the current moment, β is a parameter that obeys a normal distribution and is used for step size control, C is a random number within [−1,1], D is a constant that avoids a denominator of 0, $f_{i}$ is the value of the fitness function, $f_{b}$ is the current optimal fitness value, $f_{w}$ is the current worst fitness value, and $u_{best}$ indicates that the population is centred and secure within a certain range around it. When $f_{i}>f_{g}$, the sparrows at the edge of the population are aware of the risk, and when $f_{i}<f_{g}$, it indicates that the sparrows in the middle of the population are moving closer to their peers.

The minimum mean squared error (MSE) between the prediction set and the validation set is taken as the condition for the iterative termination of the SSA algorithm in order to find the optimal parameters, as shown in Equation (14).

(14) $$\mathrm{fitness}=\min(MSE)=\min(\frac{1}{n}\sum_{t=1}^{n}{\left(\hat{x_{t}}-x_{t}\right)}^{2})$$

where $\hat{x_{t}}$ is the predicted value of the moment t obtained after network training, $x_{t}$ is the true value of moment t in the validation set, and n is the length of the time series.

The network model searches for a set of hyperparameters that have the optimal fitness function to minimise the training error of the network based on the iterative variation in the MSE. The smaller the value of the MSE is, the better the predictive performance is, indicating that the model has a higher degree of accuracy.

**Figure 9:**
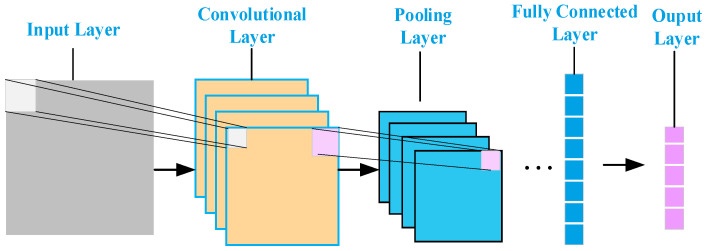
CNN model structure flow.

A CNN is a deep feed-forward neural network structural model, designed and developed based on inspiration from biological visual nerves [35,36], and the model of its basic constituent unit structure is shown in Figure 9.

In this paper, the DenseNet network structure is used as the CNN backbone network to extract the features of the input data. DenseNet adopts multiple DenseBlock connections, 2D convolution is selected, the dense connection between the convolutional layers is established by the feature cascade within each DenseBlock, and the main convolutional unit within the DenseBlock module is the BN-ReLU-Conv cascade, where BN (batch normalisation) is the batch normalisation layer, ReLU (Rectified Linear Unit) is the linear rectification activation layer, and Conv is the convolutional layer. The LSTM neural network goes through an input stage, a selective memory stage, and an output stage. Its CNN-LSTM neural network prediction model flow is as follows:

The convolutional layer is applied to the 2D input by sliding the convolution layer to create a dot product using the input matrix and the convolution kernel, as shown in Equation (15).

(15) S(i,j)=(I∗K)(i,j)=∑m∑nI(i−m,j−n)K(m,n)

where I is the original data input matrix and K is the convolution kernel.

In order to solve the problem of size reduction of the output matrix of the data input data matrix I after the operation of the convolutional layer, data padding (padding) is applied to the matrix before the input convolutional layer. Let the size of the matrix before convolution be n×n and the size of the convolution kernel be k×k. The amplitude of the padding is set to $\frac{k-1}{2}$. Then, the output of the convolved matrix is $\frac{\left(n-k+2\frac{k-1}{2}\right)}{1}+1=n$. Ensure that the size of the matrix before and after the convolution remains the same size.

The output matrix of each layer is the feature matrix, and the area that is covered by each element in the window of the convolution kernel of the original input matrix is referred to as the sensory field, as shown in Equation (16).

(16) $$RF_{i}=(RF_{i+1}-1)\times s_{i}+K_{i}$$

where $s_{i}$ and $K_{i}$ are the convolutional kernel window’s shift step and the convolutional kernel’s size for the corresponding layer i, respectively.

Pooling layer: After the data pass through the pooling layer, the pooling layer performs a pooling operation on the data and uses the statistical characteristics of the pooling window area, such as the maximum value or the average value, to represent the value of the entire pooling window, which is essentially a down sampling of the input data and reduces the size of the data. This can reduce the complexity of the feature computation and prevent overfitting during model training, and it enhances the robustness of the network.

The Flat Layer collapses the spatial dimension of the input into a channel dimension, facilitating its transition to a fully connected layer.

The fully connected layer comes after the convolutional layer and the pooling layer and is mainly used to map the learned features to the sample space and retain the complexity of the model to some extent.

Output layer: the output is performed using the softmax function (MATLAB R2022b), as shown in Equation (17).

(17) $$y_{i}=\frac{e_{zi}}{\sum_{i=1}^{n}e_{zi}}$$

A linear function was used to fit the predictions, as shown in Equation (18).

(18) $$y_{i}=\sum_{m=1}^{M}w_{im}x_{m}$$

The learning method used is the Stochastic Gradient Descent (SGD), with the following basic steps:

(1) Initialise the parameters and weights of all the convolution kernels in the network.
(2) Input the data and perform the forward steps (convolution, activation, pooling, and fully connected forward propagation).
(3) Calculate the output layer error.
(4) Calculate the gradient of the error relative to the weights using the backpropagation algorithm, and reduce and update the parameters and weights of all convolutional kernels using gradient descent. Return to step (3) to calculate the error of the output layer and keep looping until the iteration stops by satisfying the limit difference.

**Figure 10:**
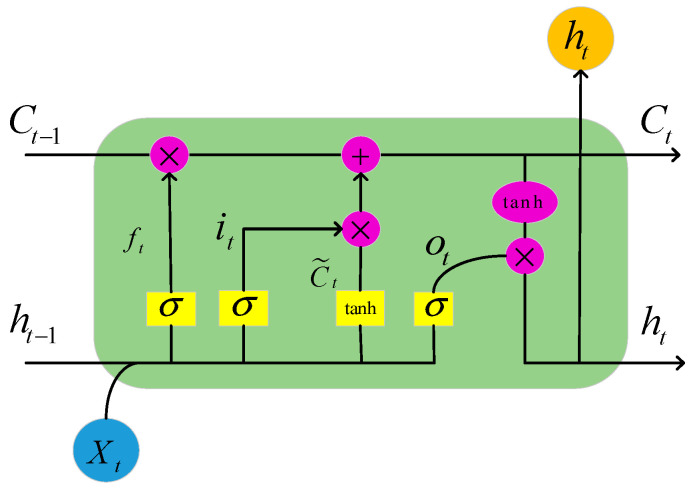
LSTM cell structure.

LSTM is a kind of neural network with a unique time series processing ability in order to prevent the problem of gradient disappearance and the explosion of a recurrent neural network, the core of which lies in the forgetting gate, input gate, and output gate, which is able to make full use of the historical time series data and capture the temporal features in the data, as well as selectively retaining and forgetting the information to predict the future data more accurately [37,38,39,40]. The structure of its network model unit is shown in Figure 10.

Building of LSTM feature learning layer: Learning the correlation between the time series and the sequence data, the features that were extracted from the CNN are fed into the LSTM layer for learning.

**Figure 11:**
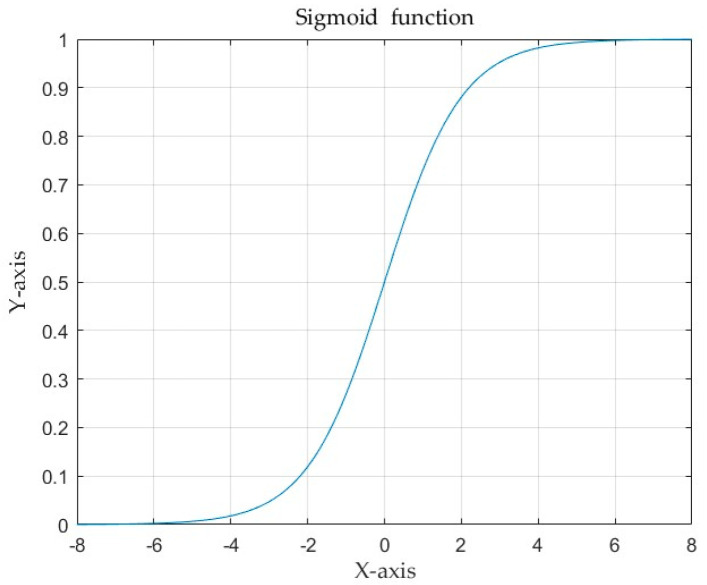
Sigmoid function graph.

Input phase: Selective forgetting of information from the previous moment’s input message is performed [41]. Whether the input is forgotten or not is controlled by the forgetting gating $f_{t}$, which controls the previous moment’s cell state, $C_{t-1}$. It is obtained by multiplying the splice vector $\left[h_{t-1},x_{t}\right]$ of the hidden state $h_{t-1}$ of the previous moment and the input value $x_{t}$ of the current moment by means of the weight matrix, followed by the bias term $b_{f}$, and then by an activation function σ; the function is shown in Figure 11. σ is the control gate with a value range of 0 to 1, which is used to forget and retain the information, and $W_{f}$ represents the weighting matrix for forgetting stages $h_{t-1}$ and $x_{t}$.

(19) $$\sigma (x)=\frac{1}{1+e^{-x}}$$

(20) $$f_{t}=\sigma (W_{f}\cdot [h_{t-1},x_{t}]+b_{f})$$

$f_{t}⊗C_{t-1}$ represents the forgotten information from the previous state $C_{t-1}$.

**Figure 12:**
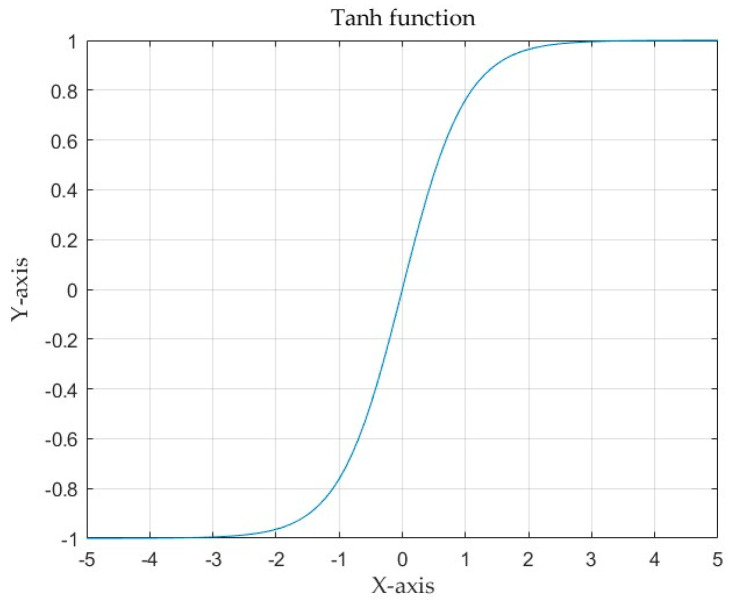
Tanh function graph.

The selective memory stage decides to selectively “remember” the input information at the current moment, enhancing the memory of important information and reducing the memory of unimportant information [42], while generating candidate cell states through the tanh function. The tanh function is shown in Figure 12 and is used to prevent the gradient from exploding or vanishing. $i_{t}$ denotes the input gating, and ${\tilde{C}}_{t}$ determines the information enhancement.

(21) $$\tanh(x)=\frac{e^{x}-e^{-x}}{e^{x}+e^{-x}}$$

(22) $$i_{t}=\sigma (W_{i}\cdot [h_{t-1},x_{t}]+b_{i})$$

(23) $${\tilde{C}}_{t}=\tanh(W_{c}\cdot [h_{t-1},x_{t}]+b_{c})$$

$z_{t}⊗i_{t}$ represents the information that is enhanced by the current input content.

The outcome of passing the next node is obtained by adding the results that were obtained in the forgetting and selective memory stages (see Equation (24)).

(24) $$C_{t}=f_{t}⊗C_{t-1}+z_{t}⊗i_{t}$$

The output phase determines the output information of the current cell state [43]. The sigmoid layer is run first to compute the output gate, and then, the output cell state $C_{t}$ that passed to the next node is multiplied by the tanh activation function to multiply the original output information pair-by-pair to obtain the final output (see Equation (25)) and the hidden state (see Equation (26)).

(25) $$o_{t}=\sigma (W_{o}\cdot [h_{t-1},x_{t}]+b_{o})$$

(26) $$h_{t}=o_{t}⊗\tanh C_{t}$$

The final output is the data that remains after the SSA-CNN-LSTM model’s prediction to obtain the surface deformation prediction value for the time series.

### 3.3. Indicators for Predictive Modelling Evaluation

In order to verify the validity of the model and measure the accuracy of the model fitting, the MAE, RMSE, MAPE, and correlation coefficient were selected as the model evaluation metrics for assessing the prediction accuracy of the SSA-CNN-LSTM model.

(27) $$MAE=\frac{1}{N}\sum_{i=1}^{N}\left|y_{i}-\tilde{y_{i}}\right|$$

(28) $$RMSE=\sqrt{{\sum_{i=1}^{N}\left(y_{i}-\tilde{y_{i}}\right)}^{2}/N}$$

(29) $$MAPE=\frac{1}{N}\sum_{i=1}^{N}\left|\frac{y_{i}-\tilde{y_{i}}}{y_{t}}\right|$$

(30) $$R^{2}=1-\frac{\sum_{i=1}^{N}{\left(y_{i}-\tilde{y_{i}}\right)}^{2}}{\sum_{i=1}^{N}{\left(y_{i}-\bar{y_{i}}\right)}^{2}}$$

In the above formulas, N is the number of data points, $y_{i}$ is the measured value, $\tilde{y_{i}}$ is the predicted value, and $\bar{y_{i}}$ is the average value.

## 4. Results

### 4.1. Analysis of Natural Influences and Deformation Results

**Figure 13:**
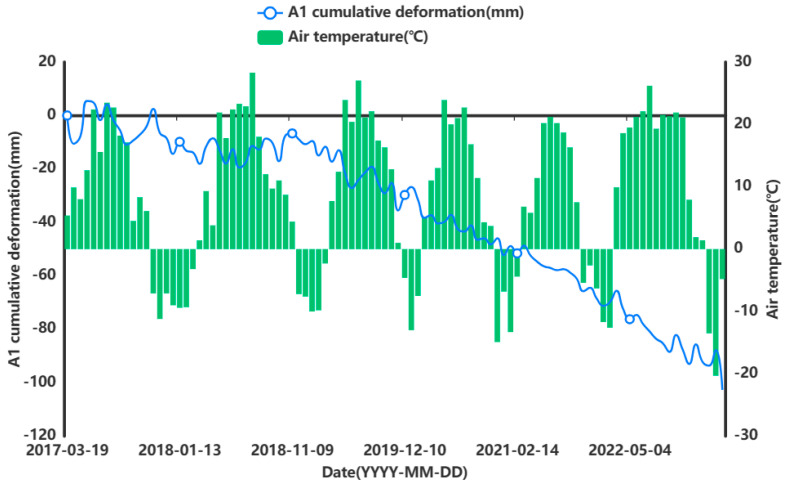
Relationship between air temperature and surface deformation.

**Figure 14:**
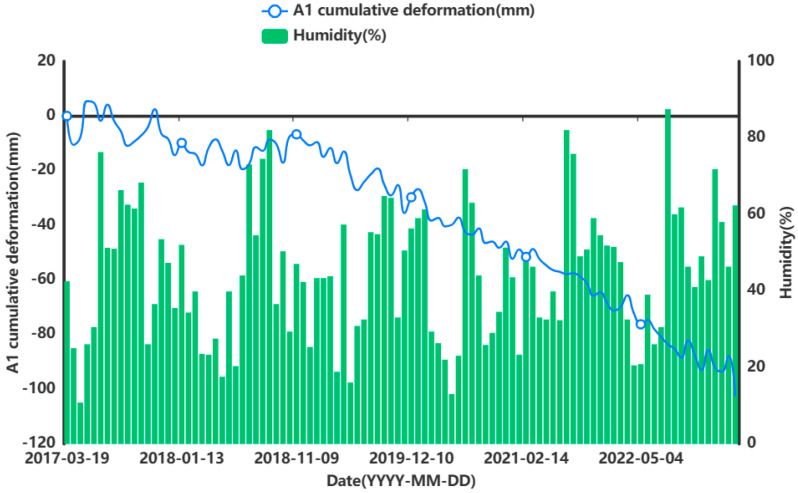
Relationship between humidity and surface deformation.

**Figure 15:**
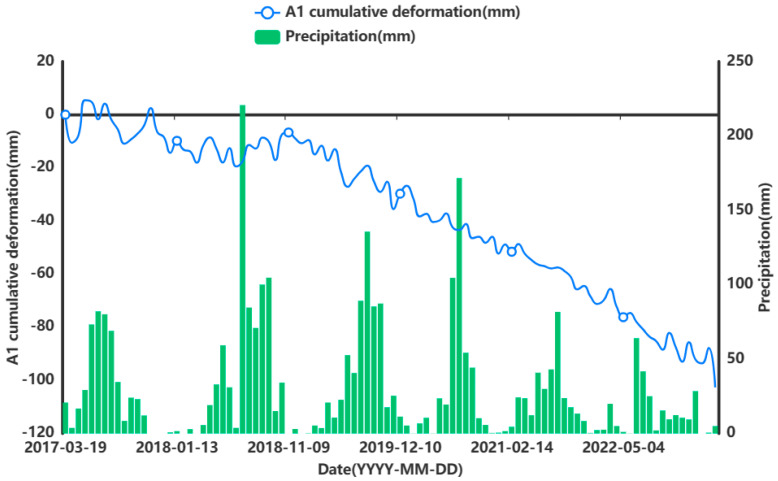
Relationship between precipitation and surface deformation.

**Figure 16:**
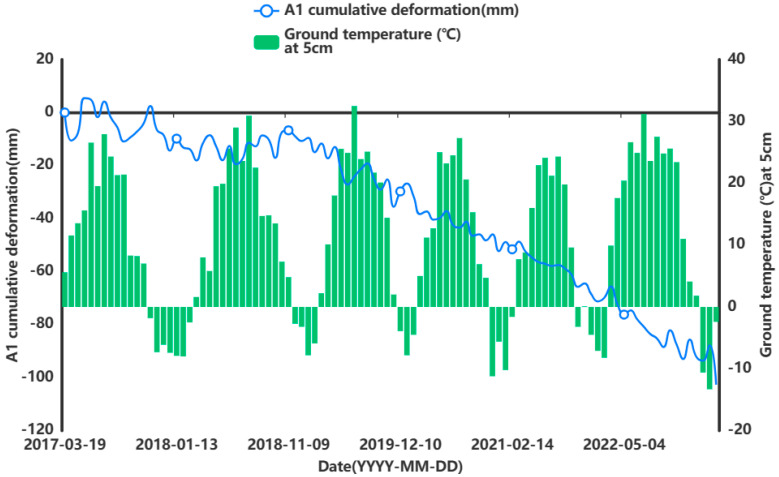
Relationship between ground temperature and surface deformation at 5 cm below the surface.

**Figure 17:**
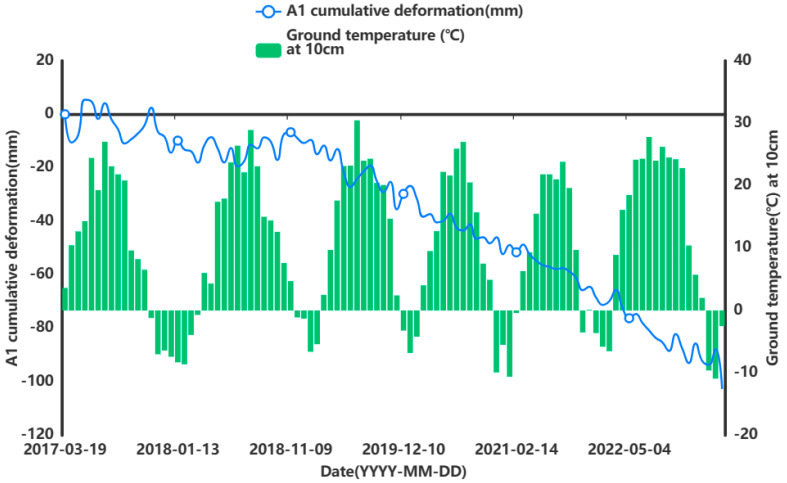
Relationship between ground temperature and surface deformation at 10 cm below the surface.

**Figure 18:**
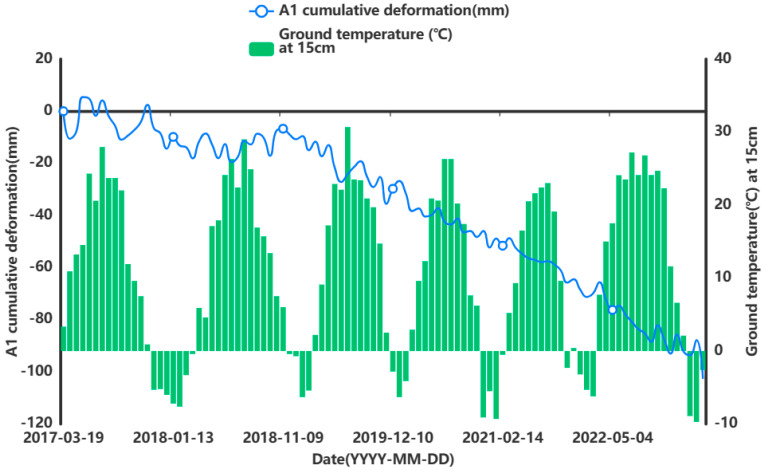
Relationship between ground temperature and surface deformation at 15 cm below the surface.

In order to facilitate the subsequent prediction study of the settlement points in the severe settlement area, only A1 is used here as an example of grey correlation analysis of the analytical feature point. A grey correlation analysis was performed between the obtained influencing factors of air temperature (°C), humidity (%), precipitation (mm), and ground temperature (°C) at 5 cm, 10 cm, and 15 cm below the ground surface and the cumulative shape variable (mm) of the feature point of A1. Figure 13, Figure 14, Figure 15, Figure 16, Figure 17 and Figure 18 represent the relationship between air temperature, humidity, precipitation, and ground temperature (at 5 cm, 10 cm, and 15 cm below the ground surface) and cumulative deformation, respectively.

**Figure 19:**
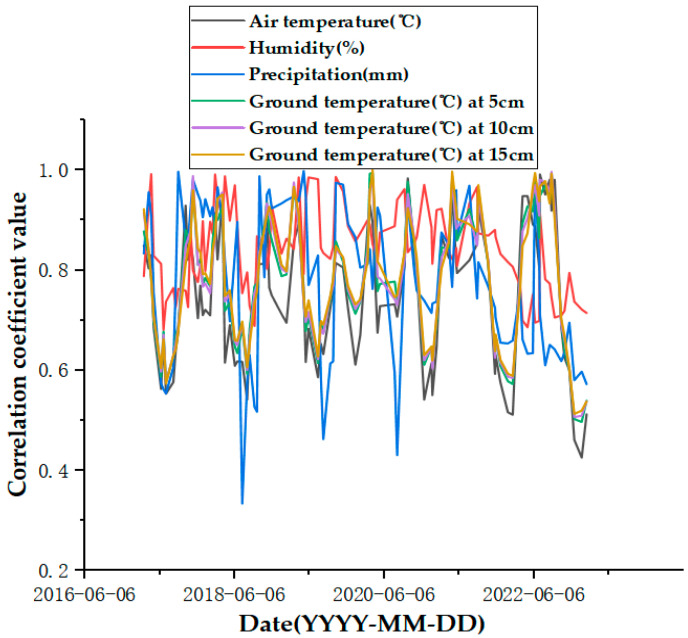
Plot of correlation coefficients between influencing factors and surface deformation.

As can be seen from Figure 13, Figure 14, Figure 15, Figure 16, Figure 17, Figure 18 and Figure 19, air temperature, humidity, precipitation, and ground temperature at 5 cm, 10 cm, and 15 cm below the surface correlate strongly with surface deformation. The deformation of the ground surface occurs in correlation with the above-combined factors, and the deformation accumulates over the time frame of the study to form ground subsidence.

The grey correlation analysis of the A1 feature points led to the following conclusions: the correlation between humidity and cumulative deformation was 0.841; the correlation between the ground temperature at 15 cm below the surface and cumulative deformation was 0.788; the correlation between the ground temperature at 10 cm below the surface and cumulative deformation was 0.785; the correlation between the ground temperature at 5 cm below the surface and cumulative deformation was 0.783; the correlation between precipitation and cumulative deformation was 0.780; and air temperature had a correlation of 0.748 with cumulative deformation. The largest correlation with cumulative deformation was that of humidity, and the smallest correlation with cumulative deformation was that of air temperature. The grey correlation statistics between each influencing factor and surface deformation are shown in Table 4.

The grey correlation analysis was applied to demonstrate that these factors contribute to the deformation of urban surfaces and that it is feasible to use these factors as input data for subsequent prediction studies. The ranking of the grey correlation coefficients demonstrates the degree of influence of the influencing factors on the deformation of the study site during the study period—the larger the coefficient is, the greater the impact of the factor is, and vice versa.

### 4.2. Results and Analyses of Cumulative Surface Deformation Predictions

As can be seen in Figure 5, the surface sedimentation in the urban area of Hohhot City is more significant, of which the western and northern areas within the study area are more obviously affected by sedimentation, and some of the eastern areas are experiencing sedimentation; on the whole, the regional sedimentation situation is relatively stable. In the grey correlation analysis, it was proven that these factors do affect surface deformation. In order to verify the validity and accuracy of the SSA-CNN-LSTM neural network prediction model, the three serious settlement points A1, B1, and C1 (which have been marked in Figure 5) in the urban area of Hohhot were selected as the research objects to predict their surface deformation in the short term. The detailed information of the three locations is shown in Table 5.

After prediction by the SSA-CNN-LSTM neural network model, the value of the fitness function is minimized by changing the number of iterations of the SSA optimization algorithm to be as close as possible to its true time series value.

**Figure 20:**
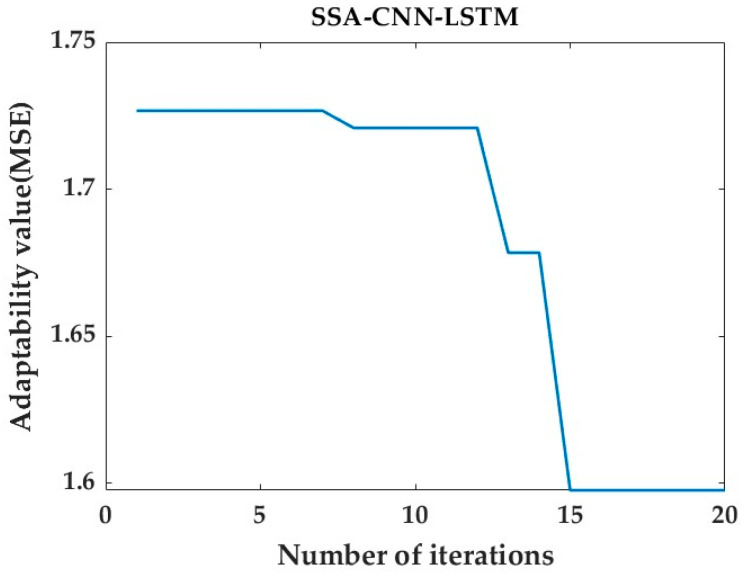
Plot of SSA fitness function for A1 prediction.

**Figure 21:**
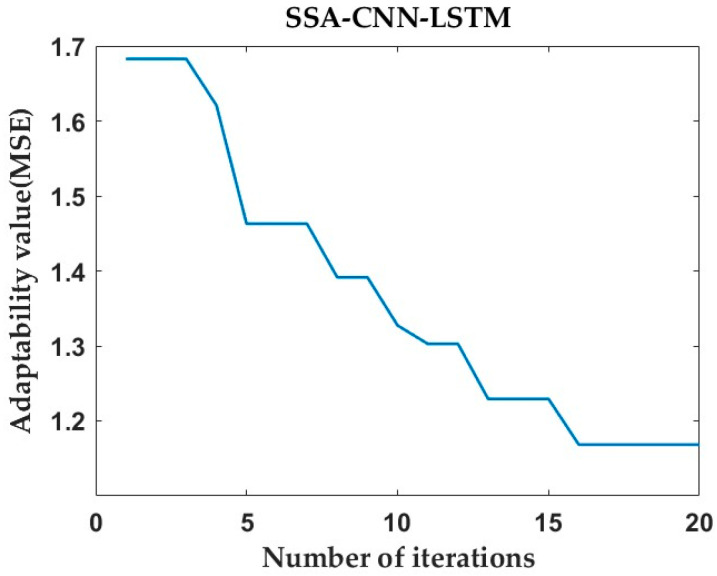
Plot of SSA fitness function for B1 prediction.

**Figure 22:**
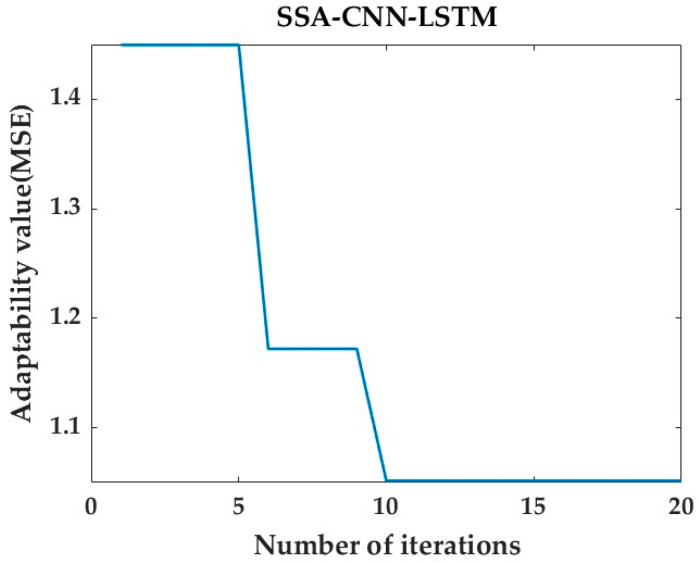
Plot of SSA fitness function for C1 prediction.

Then, after tuning the parameters, the optimal fitness function values for the predictions at positions A1, B1, and C1 are 1.597, 1.168, and 1.053, respectively. Figure 20, Figure 21 and Figure 22 represent the process of change in the fitness function for a maximum number of iterations of 20.

**Figure 23:**
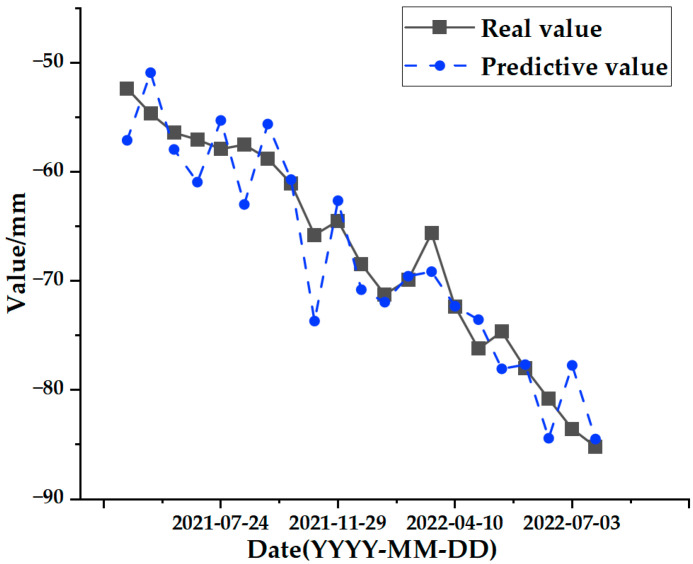
Test set comparison results for A1.

**Figure 24:**
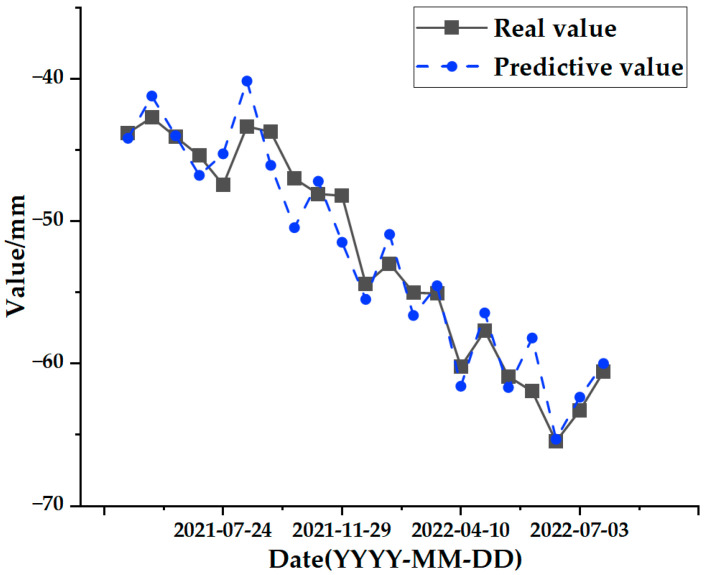
Test set comparison results for B1.

**Figure 25:**
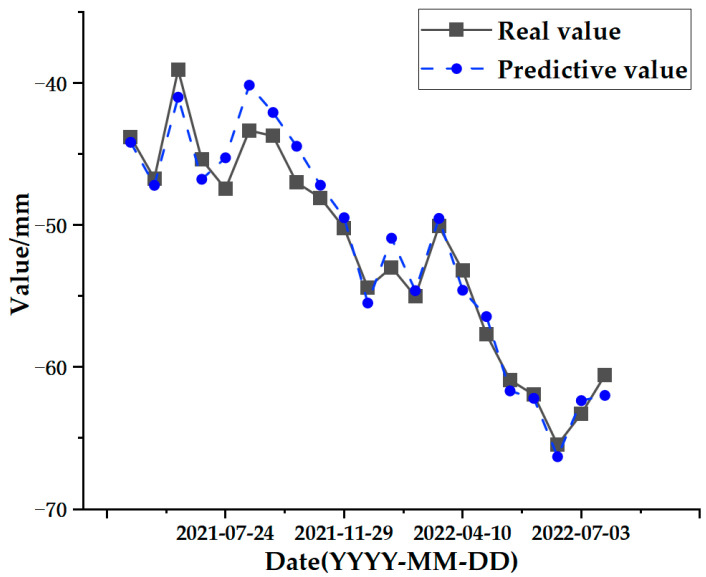
Test set comparison results for C1.

**Figure 26:**
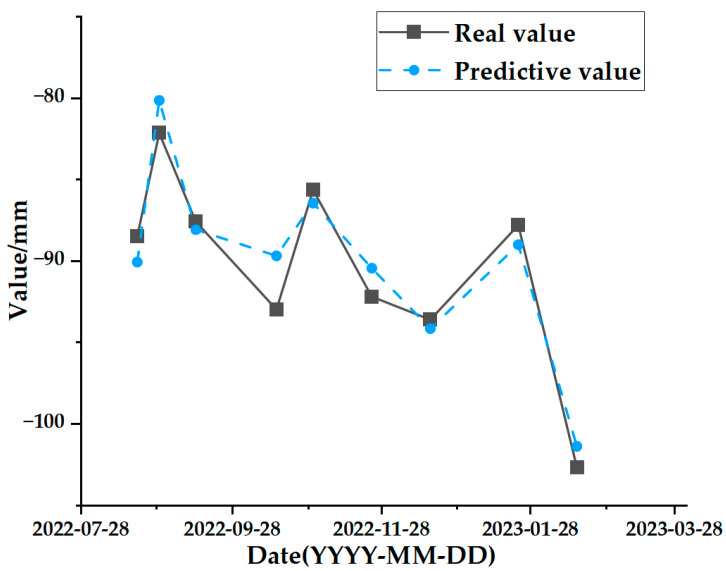
Validation set comparison results for A1.

**Figure 27:**
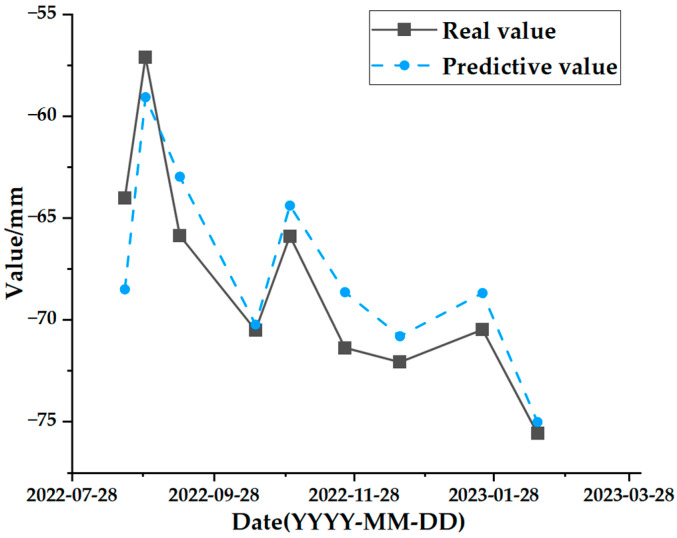
Validation set comparison results for B1.

**Figure 28:**
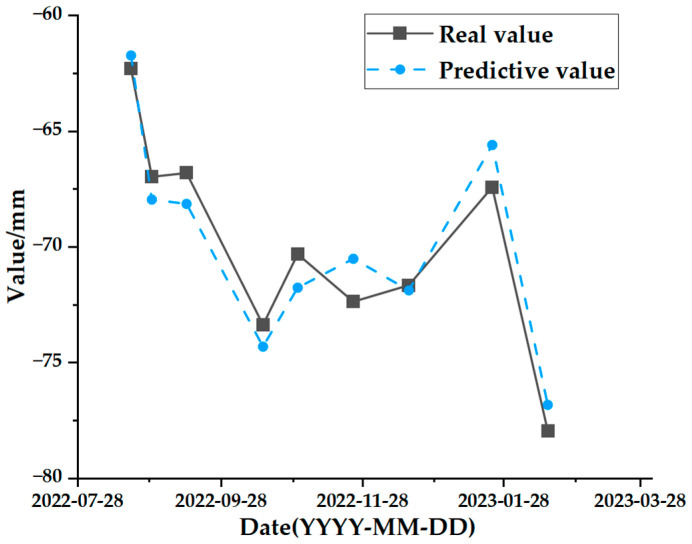
Validation set comparison results for C1.

The test set comparison results of the time series deformation values of the three feature points after being processed by the SSA-CNN-LSTM neural network prediction model are shown in Figure 23, Figure 24 and Figure 25, respectively. The results of the validation set comparison of the time series deformation values for each of the three feature points are shown in Figure 26, Figure 27 and Figure 28. Table 6 represents the comparison of the test set results for the three locations of A1, B1, and C1, and the specific results of the evaluation indexes of the SSA-CNN-LSTM neural network prediction model are shown in Table 7.

Comprehensively analyzing the above graphical data, the SSA-CNN-LSTM neural network prediction model is proven to be applicable to short-term multi-influence prediction research on urban surface deformation, and the prediction accuracy is significantly improved, the size of errors is reduced dramatically, and the model prediction is basically the same as the original time series surface deformation values.

### 4.3. Comparative Validation of the Results of Different Prediction Models

In order to further prove that the prediction accuracy of the SSA-CNN-LSTM neural network prediction model that is proposed in this paper is significantly improved, the traditional multilayer perceptron (MLP) and back propagation (BP) neural network models are used to compare and analyse the advantages and disadvantages of the prediction performance of the three methods within the SSA-CNN-LSTM method. The MLP model is a feed-forward neural network that consists of nodes in at least three layers: an input layer, one or more hidden layers, and an output layer. The MLP model is one of the most commonly used neural networks in supervised learning, and it is capable of learning nonlinear mapping relationships between inputs and outputs. The BP neural network model is a kind of multilayer feed-forward network that is trained according to the error back propagation algorithm, and it is one of the most widely used neural network models at present. The learning rule of the BP neural network is to use the most rapid descent method to continuously adjust the weights and thresholds of the network by means of back propagation to minimize the sum of error squares of the network.

**Figure 29:**
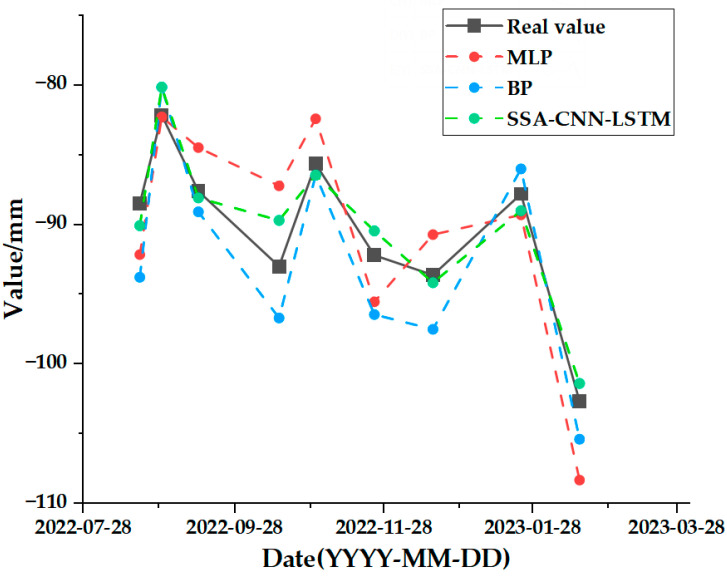
Comparison of prediction results of different methods for A1.

**Figure 30:**
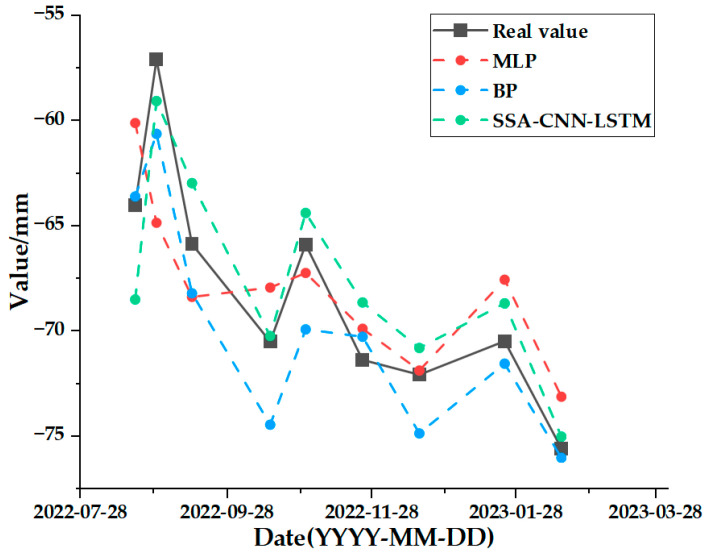
Comparison of prediction results of different methods for B1.

**Figure 31:**
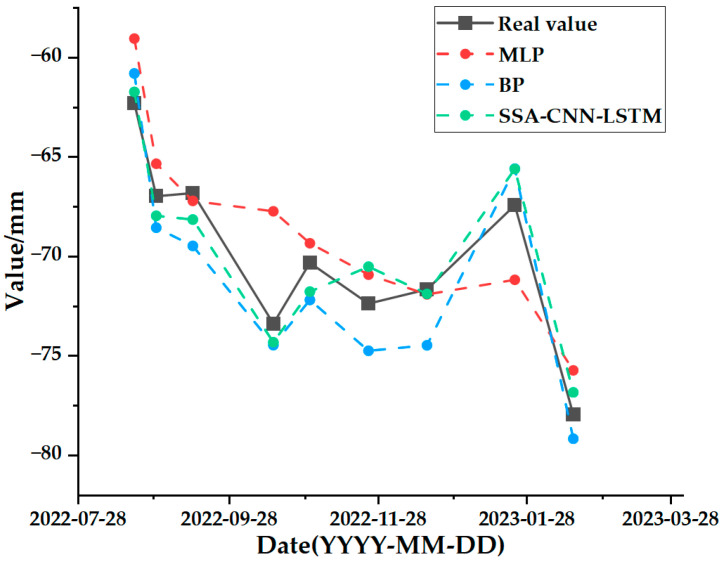
Comparison of prediction results of different methods for C1.

Figure 29, Figure 30 and Figure 31 represent the comparative analyses of the prediction results of feature points A1, B1, and C1 using different methods, respectively. Table 8, Table 9 and Table 10 show the prediction evaluation indexes of A1, B1, and C1 using the different methods.

According to the results shown in the above charts, the prediction results of the SSA-CNN-LSTM neural network model that is proposed in this paper are much higher than those of the traditional methods of the MLP and BP neural networks for the feature points of A1, B1, and C1, and they are also much closer to the real values.

## 5. Discussion

The experimental results show that the time series surface deformation prediction is of great practical significance for urban planning, construction, and development, and a multifactorial analysis of surface deformation provides a higher accuracy for time series surface deformation predictions.

In this paper, the SSA-CNN-LSTM neural network prediction model is established by combining the advantages of the SSA’s strong learning ability and fast convergence speed and the CNN’s strong feature extraction ability. The validity and accuracy of the model are then verified by using multiple influencing factors and surface deformation data, and the model is able to actively learn the complex feature representations of the data. In addition, the LSTM network is suitable for dealing with the time series data and is able to capture the long-term dependent relationships, while the CNN is able to handle the spatial features. This combination makes the model highly capable of handling spatio-temporal data. A good prediction accuracy is achieved.

However, there are also problems, such as insufficient influencing factors and changes in the parameters of the neural network model due to data changes. Therefore, when using the SSA-CNN-LSTM model for predictions at different locations, problems such as overfitting that cannot be avoided in the traditional method are compensated for by debugging each parameter in order to improve the prediction accuracy and efficiency of the model. From the above experimental results (Figure 20, Figure 21, Figure 22, Figure 23, Figure 24, Figure 25, Figure 26, Figure 27 and Figure 28 and Table 6 and Table 7), it can be seen that the SSA-CNN-LSTM model shows good prediction results, which are close to the original actual data values.

In order to further verify the prediction accuracy of the SSA-CNN-LSTM neural network model, the MLP and BP neural network models are selected for comparative analysis, as shown in the graphs of the prediction results of different methods (Figure 29, Figure 30 and Figure 31 and Table 8, Table 9 and Table 10), which clearly illustrate that the prediction accuracy of the model that is proposed in this paper is superior to those of the traditional MLP and BP neural network models.

Therefore, the SSA-CNN-LSTM neural network prediction model can be used for time series surface deformation prediction, which will provide an early warning for the safe operation of cities and also make an important contribution to the working lives of residents and to environmental protection.

This research method is effective for the prediction of surface deformation in urban areas, but the prediction accuracy is significantly affected by the combination of man-made and natural factors, and in this paper, due to the data, only the influence of the main natural and meteorological factors is considered. In future research, investigators may consider adding human factors such as population and buildings to the neural network prediction model to improve its prediction accuracy.

## 6. Conclusions

This paper takes Hohhot City in the Inner Mongolia Autonomous Region as its study area, and based on the Sentinel-1A descending orbit image data and the relevant data provided by the meteorological department, it makes full use of the high-precision deformation monitoring results of the SBAS-InSAR time series InSAR technology and obtains deformation prediction values of high precision and high spatial resolution. The study uses grey correlation analysis to explore the correlation between the original data variables, determine the degree of influence of various factors on surface deformation, take the meteorological influences as the input of the neural network prediction model, and establish the SSA-CNN-LSTM neural network prediction model for predicting and analysing the surface deformation of urban areas that are affected by a variety of natural factors. The following conclusions are drawn:

(1) The SBAS-InSAR technique can effectively obtain time series surface deformation information in the study area. The surface subsidence in the main urban area of Hohhot is more obvious in the western and northern regions within the study area, and the eastern region shows partial subsidence. On the whole, the regional subsidence is relatively stable. Combined with the grey correlation analysis method, it was verified that the deformation area was correlated with the natural factors of air temperature, humidity, precipitation, and ground temperature (5 cm, 10 cm, 15 cm), and the analysis also provided some support for the subsequent integration of the influencing factors into the prediction study.
(2) Based on the time series SBAS-InSAR surface deformation results, the SSA-CNN-LSTM neural network prediction model was established. The most serious point locations of subsidence in the three regions were selected, and the correlation coefficients between the prediction results of the SSA-CNN-LSTM neural network and the actual InSAR results are all above 0.96. At the same time, comparative analyses were performed using the traditional methods of the MLP and BP neural network models to evaluate the prediction results for the different point locations. If the prediction accuracy of the SSA-CNN-LSTM neural network prediction model is superior to the two traditional models, it can be intuitively concluded that the SSA-CNN-LSTM neural network is better at assessing the relationship between the influencing factors and the dependent variables in the data and that it can more accurately predict the surface deformation within a short period of time at the point prediction scale, which not only improves the performance and generalization ability of the neural network prediction model but also improves the robustness of the entire neural network prediction model.

## Figures and Tables

**Table 1:** Main parameters of the Sentinel-1A data used in this study.

Parameter	Value
Pass direction	Descending
Beam mode	IW
Polarization	VV
Wave band	C
Wavelength/cm	5.6
Number of images	100
Monitored period	19 March 2017–2 February 2023

**Table 2:** SSA parameter settings.

Parameter	Value
Warning value, ST	0.7
Percentage of discoverers, PD	0.4
Awareness of dangerous specific sparrow gravity, SD	0.2
Number of optimisation parameters	3

**Table 3:** Main parameter settings for SSA-CNN-LSTM.

Parameter	Value
Input dimension	6
Output dimension	1
Sample batch processing	128
Optimizer	Adam
Number of iterations	500
Loss	Mean Absolute Error

**Table 4:** Grey correlation analysis statistics.

Influencing Factors	Grey Correlation	Order of Significance
Humidity (%)	0.841	1
Ground temperature (°C) at 15 cm	0.788	2
Ground temperature (°C) at 10 cm	0.785	3
Ground temperature (°C) at 5 cm	0.783	4
Precipitation (mm)	0.780	5
Air temperature (°C)	0.748	6

**Table 5:** Marker location details.

Name	Sinking Centre	Latitude and Longitude/°	Maximum Cumulative Sedimentation/mm
A1	Near the intersection of West Genghis Khan Street and North Bayannur Road	40°50′32.40″ N, 111°37′21.57″ E	−102.674
B1	Near Hugang South Road	40°47′19.21″ N, 111°37′47.97″ E	−75.572
C1	Near Shuangdaishe neighbourhood	40°47′34.81″ N, 111°42′36.56″ E	−77.949

**Table 6:** Validation set comparisons.

Validation Set	A1		B1		C1	
	Real Value	Predictive Value	Real Value	Predictive Value	Real Value	Predictive Value
2022-08-20	−88.489	−90.068	−64.001	−68.489	−62.290	−61.725
2022-08-29	−82.139	−80.134	−57.081	−59.057	−66.970	−67.955
2022-09-13	−87.593	−88.073	−65.854	−62.960	−66.809	−68.143
2022-10-16	−92.983	−89.690	−70.497	−70.232	−73.374	−74.314
2022-10-31	−85.635	−86.440	−65.875	−64.377	−70.316	−71.766
2022-11-24	−92.189	−90.441	−71.365	−68.628	−72.371	−70.513
2022-12-18	−93.608	−94.151	−72.072	−70.795	−71.659	−71.878
2023-01-23	−87.794	−88.997	−70.472	−68.676	−67.418	−65.594
2023-02-16	−102.674	−101.388	−75.572	−75.014	−77.949	−76.832

**Table 7:** SSA-CNN-LSTM model evaluation indicators.

Characteristic Point	MAE	MSE	RMSE	$R^{2}$
A1	5.398	3.716	1.928	0.973
B1	4.475	2.672	1.635	0.982
C1	4.604	3.584	1.893	0.965

**Table 8:** A1 indicators for evaluating forecast results.

Method	MAE	RMSE	MAPE	$R^{2}$
MLP	3.26	3.66	3.53%	0.62
BP	2.88	3.20	3.16%	0.71
SSA-CNN-LSTM	1.44	1.66	1.59%	0.92

**Table 9:** B1 indicators for evaluating forecast results.

Method	MAE	RMSE	MAPE	$R^{2}$
MLP	2.80	3.45	4.33%	0.61
BP	2.19	2.59	3.29%	0.78
SSA-CNN-LSTM	1.94	2.29	2.93%	0.83

**Table 10:** C1 indicators for evaluating forecast results.

Method	MAE	RMSE	MAPE	$R^{2}$
MLP	2.18	2.74	3.13%	0.64
BP	1.88	1.97	2.70%	0.82
SSA-CNN-LSTM	1.14	1.25	1.64%	0.93

## Data Availability

No new data were created in this study. Data sharing is not applicable to this article.
